# Identification and validation of reference genes for normalization of gene expression analysis using qRT-PCR in *Megalurothrips usitatus* (thysanoptera: thripidae)

**DOI:** 10.3389/fphys.2023.1161680

**Published:** 2023-04-18

**Authors:** Qingfang Hou, Linlin Yuan, Haifeng Jin, Han Yan, Fen Li, Shaoying Wu

**Affiliations:** ^1^ Sanya Nanfan Research Institute, Hainan University, Sanya, China; ^2^ School of Plant Protection, Hainan University, Haikou, China

**Keywords:** *Megalurothrips usitatus*, light, qRT-PCR, reference genes, stability

## Abstract

**Introduction:**

Gene expression analysis by reverse transcription quantitative polymerase chain reaction (qRT-PCR) has been widely used in research including insects. The selection of appropriate reference genes is the key to obtaining accurate and reliable results from qRT-PCR. However, studies on the expression stability of reference genes in *Megalurothrips usitatus* remain lacking.

**Methods:**

In this study, qRT-PCR was used to analyze the expression stability of candidate reference genes in *M. usitatus*. The expression levels of six candidate reference gene transcription of *M. usitatus* were analyzed. GeNorm, NormFinder, BestKeeper, and ΔCt were used to analyze the expression stability of *M. usitatus* treated with biological factors (developmental period treatment) and abiotic factors (light, temperature, insecticide treatment, respectively). Comprehensive stability ranking of candidate reference genes was recommended by RefFinder.

**Results and Discussion:**

Results showed that ribosomal protein S (*RPS*) was the most suitable expression in insecticide treatment. Ribosomal protein L (*RPL*) was the most suitable expression at developmental stage and light treatment, whereas elongation factor was the most suitable expression in temperature treatment. RefFinder was used to comprehensively analyze the above four treatments, and the results showed that *RPL* and actin (*ACT*) showed high stability in each treatment. Therefore, this study identified these two genes as reference genes in the qRT-PCR analysis of different treatment conditions of *M. usitatus*. Ourfindings will be beneficial for improving the accuracy of qRT-PCR analysis for future functional analysis of the target gene expression in *M. usitatus*.

## Highlights

1. The stable reference genes were firstly analyzed in *M. usitatus* under different conditions.
2. This study confirmed appropriate combination of reference genes under different factors.
3. Robust procedures were established to quantify gene expression in *M. usitatus.*
4. The stable internal reference genes of *M. usitatus* at different developmental stages and under different treatment conditions are not completely consistent.
5. The RPL and ACT gene was considered as the suitable reference genes for *M. usitatus.*

## 1 Introduction

Reverse transcription quantitative polymerase chain reaction (qRT-PCR) can be used in qualitative research, such as heterozygote and homozygote identification, by monitoring the entire process of PCR in real time through fluorescence signals. Analysis of single nucleotide polymorphisms can also be used in quantitative studies, such as gene copy number counting, absolute quantitative studies of virus and pathogen analysis, mRNA expression analysis, and relative quantitative studies of microarray results verification (Kubis et al., 2013; Zeng et al., 2014). As a revolutionary leap in the field of molecular biology, qRT-PCR has been applied to gene expression and clinical diagnosis or transcriptome analysis in plants, animals (Zhao et al., 2011; Zeng et al., 2014), and microorganisms due to its advantages of simple operation, high sensitivity, good repeatability, and high throughput. It has also been reported in insect transcriptome analysis or reference gene screening (Kim et al., 2013).

As a reference for the detection of gene expression level changes, the accuracy of qRT-PCR results is often determined by the stable expression of reference genes in different cells or physiological states. The ideal reference gene is a reference gene that consistently shows transcript abundance similar to the target gene under different experimental conditions and does not coregulate with the target gene (Radonic et al., 2004). As a result, 18 S ribosomal RNA (*18* *S RRNA*), glyceraldehyde 3-phosphate glyceraldehyde dehydrogenase gene (*GAPDH*), transcription elongation factor gene (*EF*), actin gene (*ACT*), tubulin gene (*TUB*), histone gene (*H2A*), heat activator protein (*HSP*), succinate dehydrogenase B subunit (*SDHB*), ribosomal protein (*RPL/RPS*), and other constitutively expressed reference gene are often selected as reference genes because they are either involved in the basic biochemical metabolic process of the organism or the basic components of the cytoskeleton, which can be stably expressed in different cells in theory. The choice of the correct reference gene depends largely on the biological or abiotic stress being studied, both of which affect the specific expression of the reference gene. Given the influence of the internal and external environment, the ideal reference gene may not be present in all different treatments (Bustin, 2002; Folkersen et al., 2009). The expression of the ideal reference gene at different stages of development and under different experimental conditions is only relatively stable (Thorrez et al., 2008). Choosing the right reference gene is also a crucial step. However, numerous experimental results show that the expression stability of HKGs is only relatively stable in a certain range. The expression levels of the same HKGs may vary greatly in different species or different tissues of the same species, as well as under different environmental stress conditions. Previous studies have shown that at least two or three reference genes should be used to standardize gene expression data (Thellin et al., 1999; Lin and Lai, 2010). Therefore, appropriate reference genes must be identified to standardize qRT-PCR data. Furthermore, one or more reference genes that are stably expressed in specific experimental materials and conditions must be screened out.

*M. usitatus* (Thysanoptera: Thripidae) is an important pest on legume crops, causing serious damage to *V. unguiculata* crops. It can harm the whole growth period of *Vigna unguiculata.* During high-temperature and dry season, *V. unguiculata* often causes the atrophy of stem tip, leaf deformity, drop of flowers and pods, etc., which seriously affects the yield and quality of *V. unguiculata* and even completely fails to harvest. At present, *Megalurothrips usitatus* relies mainly on chemical control, and the production of pesticide residues due to the heavy use of pesticides leads to excessive pesticide residues. In *M. usitatus*, the resistance gene expression of *M. usitatus* by qRT-PCR is lacking, and the mining of reference genes remains blank. The stability and effectiveness of reference genes have not been validated, and thus might significantly affect statistical analyses and might result in false data interpretation (Lu et al., 2013). Therefore, identifying the optimal reference gene for specific conditions in *M. usitatus* is imperative.

In this study, biological factors (second instar larvae, pupa, and adults) of *M. usitatus*, abiotic factors (white, blue and ultraviolet light), temperature (4°C, 24°C, and 35°C), and insecticides (bifenthrin and emamectin benzoate) were selected to process the experimental samples. The expression levels of *EF, ACT, HSP, SDHB, RPS* and *RPL* in *M. usitatus* were detected under these treatments. GeNorm, NormFinder, BestKeeper and ΔCt were used to evaluate the expression stability of each candidate gene, and the relative stability level under representative conditions was obtained. Moreover, the reference gene combination *RPL* and *ACT*, which can be stably expressed under the four typical treatments, provided a reference basis for the subsequent gene expression study of *M. usitatus*. The results lay a foundation for the expression analysis of functional genes of *M. usitatus* and provide a reference for further study of the expression differences of *M. usitatus* under different environmental stresses.

## 2 Materials and methods

### 2.1 Insects

The relatively sensitive strain of *M. usitatus* (HNS) was provided by Professor Fan Yongmei of Hainan University. Cowpea seeds were breeded by Liaoning Vegetable Research Institute and named Liaoshu 105. The seeds were planted without exposure to any pesticide. The *M. usitatus* were fed with fresh cowpea to subsist and reproduce and the insects were reared in an artificial incubator (RDN-300B, Ningbo Yanghui Instrument Co., Ltd.); all stages were maintained at 26°C ± 1°C, 50%–60% relative humidity, with a photoperiod of 16 L: 8 D. *V. unguiculata* was cut into 8–10 cm lengths with solid ends. The cowpeas were put in a transparent tissue culture bottle with spawning tablets on the bottom, and the bottle was filled without any insecticide beans (8–10 cm), replace *V. unguiculata* every 48 h. A large square hole was cut on the top of the tissue culture bottle. A 200-mesh screen was installed and fastened with a leather band to prevent the *M. usitatus* from drilling out and escaping.

### 2.2 Sample treatments

In this study, the samples from three developmental stages (second-instar larvae, pupa, adults), three kinds of light (white, blue, and ultraviolet light), three temperatures (4°C, 24°C, and 35°C), and two concentrations of two kinds of insecticides (Bifenthrin: 678.45 mg/L and 925.353 mg/L; Emamectin Benzoate: 0.219 mg/L and 8.350 mg/L) were used to extract RNA, and then the expression level was analyzed by qRT-PCR.

In the developmental treatment group, 100 second-instar larvae, 100 one-day-old pupa, and 50 two-day-old adults (25 males and 25 females) of *M. usitatus* were separately collected for subsequent experiments.

In the light treatment group, two-day-old adults reared under normal conditions were first treated in the dark for 24 h. Then, the adults were transferred into three light conditions: blue light (10 W, 460 nm), ultraviolet light (10 W, 360 nm) and white light (10 W, 700 nm). A total of 50 adults were collected as one replicate under these treatments at 3, 6, and 9 h for subsequent experiments.

In the temperature treatment group, two-day-old adults reared under normal conditions were transferred into three temperatures (4°C, 24°C, and 35°C) for continued breeding. A total of 50 adults were collected as one replicate after 2, 6 and 12 h for subsequent experiments.

In the insecticide treatment group, two-day-old adults were treated with bifenthrin and methylamectin benzoate (Li et al., 2013), which are commonly used in field to control *M. usitatus*. The mortality of *M. usitatus* was observed at 72 h. At 24, 48, and 72 h, 50 surviving adults were collected from the concentration at 25% and 75% mortality of bifenthrin and methylamectin benzoate treatments.

The *M. usitatus* under normal feeding conditions were collected as control. Three replicates were set up for each treatment group. All treatment samples were collected in 1.5 mL enzyme-free tubes (LABSELECT, Anhui Province, China), quickly frozen in liquid nitrogen, and stored in a −80°C freezer to extract RNA.

### 2.3 Reference gene selection and primer design

Six commonly used HKGs were selected, including *ACT*, *EF*, *RPL*, *RPS*, *HSP* and *SDHB*. On the basis of *Thrips palmi* and *Frankliniella occidentalis* synthesized by Sangon Biotech (Shanghai, China), the primers were designed using Primer Premier 5.0 (Primer, Canada) software. The accession numbers of these genes and primers were listed in Table 1.

**TABLE 1:** Information of primers used for qRT-PCR.

Gene name	Gene full name	Primer sequences	Length (bp)	Amplicon size (bp)	Accession number
*ACT*	Actin	ACG​ACG​TAC​AAC​TCC​ATC​AT	125	1,451	XM_034383284
		GTA​ATC​TCC​TTC​TGC​ATC​CTG​T			
*EF*	elongation factors 1 alpha	ATG​AAC​AAG​ATG​GAC​CGT​GC	147	2,890	XM_026432438
		GTC​AAC​TCG​CAC​TTC​ACC​CAT​G			
*RPL*	ribosomal protein L	ACATCGAGCTGGGTACTG	122	757	XM_026436599
		CAC​CAC​CAT​TTA​CTG​AGC​AT			
*RPS*	ribosomal protein S	GAC​CCC​AGA​TTT​GTG​GAA​GG	103	941	XM_026436453
		TTCAGGTCGCTGGGCAAT			
*SDHB*	Succinate dehydrogenase	CTG​TAT​CTG​GGC​AGC​GAT​TG	121	2,223	JQ655740
		AGC​ACC​ATG​GGA​CCA​CTC​T			
*HSP*	Heat Shock Proteins	CAA​CGA​GAC​CGT​CAA​GTG​G	137	1,517	XM_026416122
		GCT​TGG​TGA​TGA​TGG​GGT​T			

### 2.4 Total RNA extraction and cDNA synthesis

All samples were collected in accordance with the above instructions and then used for total RNA extraction by Trizol reagent (Invitrogen, USA) following the manufacturer’s protocol. The concentration of genomic RNA was determined by Micro Drop (BIO-DL Corporation, Shanghai, China), and its integrity was detected with 1% agarose gel electrophoresis. RNA with an A260/280 ratio range of 1.8–2.1 and a 260/230 ratio >2.0 was used for the synthesis of cDNA. By using the PrimeScript RT Reagent Kit with gDNA Eraser (Takara, Dalian, China) Kit, cDNA was synthesized from total RNA in accordance with the kit’s instructions and stored at −20°C until use.

### 2.5 Construction of standard curves and qRT-PCR

qRT-PCR was performed using a TB Green Premix Ex Taq Kit (Takara, Dalian, China) containing ROX in an Applied Biosystems 7,500 Real-Time PCR System (Applied Biosystems, Foster City, CA). Reactions were performed in a 25 μL volume mixture containing 2 μL of cDNA template, 10 μL of TB Green Premix Ex Taq, 0.4 μL of upstream and downstream primers (10 μM), and 6.8 μL of sterile water (Note: *HSP* and *SDHB* genes are relatively special, and the amount of primers and sterile water was changed during the addition of samples, whereas the amount of other reagents and the total volume remained unchanged. The amount of *HSP* primer was 0.6 μL, and that of sterile water was 6.4 μL. The amount of *SDHB* primer was 0.8 μL, and that of sterile water was 6.0 μL). The thermal cycling procedure was as follows: predeformation at 95°C for 30 s, followed by 40 cycles of 95°C for 5 s and 60°C for 30 s; at the end of each PCR reaction, dissolution curves from 60°C to 95°C were analyzed for all reactions to ensure specificity of the amplified products. The reliability of the qRT-PCR results was confirmed by standard curve and melting curve analysis. Standard curves were created by using 10-fold dilution series of cDNA (1:10, 1:100, 1:1,000, 1:10000, and 1:100000) as a template for each treatment using the linear regression model (Thellin et al., 1999). The efficiencies (E) of corresponding primers used in qRT-PCR were calculated in accordance with the equation, E = (10 [−1/slope] − 1) × 100.

### 2.6 Stability analysis of reference gene expression

A web-based analysis tool, namely, RefFinder (https://www.heartcure.com.au/reffinder), was used to conduct a comprehensive assessment on the stability of the candidate. RefFinder integrated all the four software applications, including geNorm, NormFinder, BestKeeper, and compared ΔCt method. On the basis of the rankings from each program, RefFinder assigned an appropriate weight to an individual gene and calculated the geometric mean of their weights for the overall final ranking (Xie et al., 2012).

### 2.7 Validation of reference gene selection

To evaluate the validity of the optimized selection of reference genes, the expression levels of the cytochrome P450 gene (*CYP450*) were analyzed in different experimental conditions (developmental period, light, temperature, insecticide treatment). For each experimental condition, the expression profiles of the gene *CYP450* were normalized using only one reference gene (the most stable reference gene [NF1], two stable reference genes [NF1-2] and the least stable reference gene [NF6]) recommended by RefFinder. The relative expression levels of *CYP450* in different samples were calculated according to the 2^−ΔΔCt^ method (Livak and Schmittgen, 2001). All the experiments were performed in triplicate, and the results are expressed as means ± standard deviation (SD). Statistically significant differences from target gene expression are denoted by ** (*p* < 0.001) and *** (*p* < 0.0001) as determined by the *t*-test (and non-paramentric tests) analysis in GraphPad Prism 5.0 software.

## 3 Results

### 3.1 Selection of reference gene

A total of six HKGs of *ACT, EF, RPL, RPS, HSP,* and *SDHB* were selected in this study. The primer specificity for qRT-PCR was verified by melting curve analysis. All the primer pairs amplified a single PCR product with the expected sizes and sequences, showed a slope less than −3.1, and exhibited regression coefficient (*R* ^2^) and efficacy values ranging from 0.987 to 0.999 and 90.6%–109.1%, respectively (Table 2). These data indicate that the amplification efficiencies of the primers reached the standard requirements of conventional qRT-PCR.

**TABLE 2:** Primer specificity of six reference genes for qRT-PCR.

Gene name	Primer sequences	Length (bp)	Slope	Efficiency (%)	R2
*ACT*	ACG​ACG​TAC​AAC​TCC​ATC​AT	125	−3.339	99.3	0.999
	GTA​ATC​TCC​TTC​TGC​ATC​CTG​T				
*EF*	ATG​AAC​AAG​ATG​GAC​CGT​GC	147	−3.374	99.7	0.997
	GTC​AAC​TCG​CAC​TTC​ACC​CAT​G				
*RPL*	ACATCGAGCTGGGTACTG	122	−3.482	93.64	0.999
	CAC​CAC​CAT​TTA​CTG​AGC​AT				
*RPS*	GAC​CCC​AGA​TTT​GTG​GAA​GG	103	−3.120	109.1	0.996
	TTCAGGTCGCTGGGCAAT				
*SDHB*	CTG​TAT​CTG​GGC​AGC​GAT​TG	121	−3.412	96.3	0.999
	AGC​ACC​ATG​GGA​CCA​CTC​T				
*HSP*	CAA​CGA​GAC​CGT​CAA​GTG​G	137	−3.569	90.6	0.987
	GCT​TGG​TGA​TGA​TGG​GGT​T				

**FIGURE 1:**
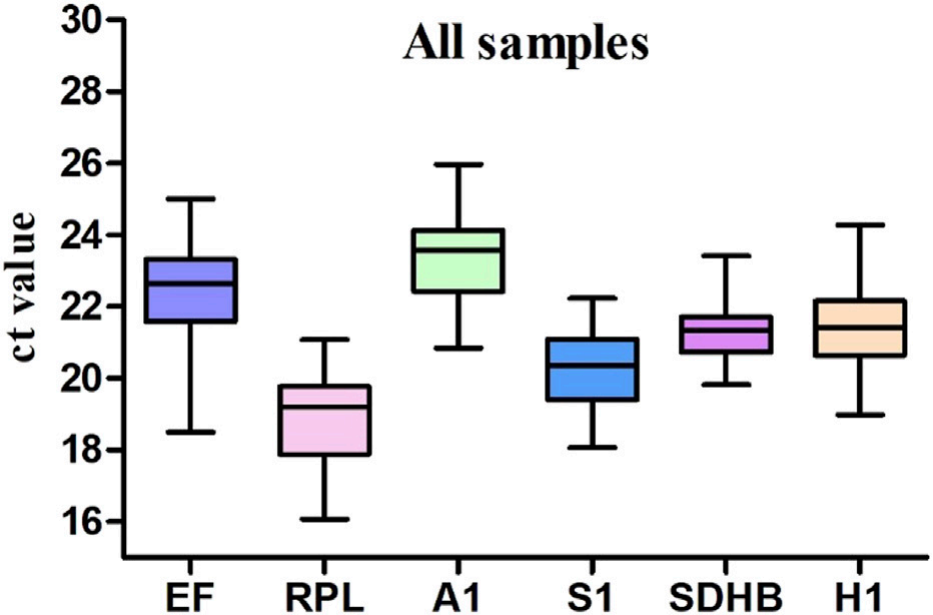
Expression profiles of reference genes in different treatment samples of *Megalurothrips usitatus.*

The Ct values in qRT-PCR provided an overview of the variation in gene expression in the samples. The distributions of the mean Ct values for each gene in all samples significantly varied. The Ct values (n = 144 samples) of the six candidate reference genes ranged from 16.86 for *RPL* to 26.079 for *HSP*. The remaining reference genes were expressed at moderate levels, with mean Ct values of 22.74911111, 19.0510809, 23.65438575, 20.4980881, 21.61548125, and 21.86929713 for *EF, RPL, RPS, ACT, SDHB,* and *HSP*, respectively (Figure 1). The significant variation in raw expression levels indicates that selecting a suitable reference gene for normalization requires confirmation of the expression stability.

### 3.2 Expression variations analysis of the six reference genes

In this study, the samples from three developmental stages (second-instar larvae, pupa, adults), three kinds of light (white, blue, and ultraviolet light), three temperatures (4°C, 24°C, and 35°C), and two concentrations of two kinds of insecticides (Bifenthrin: 678.45 mg/L and 925.353 mg/L; Emamectin Benzoate: 0.219 mg/L and 8.350 mg/L) were used to extract RNA, and then the expression level was analyzed by qRT-PCR.

**FIGURE 2:**
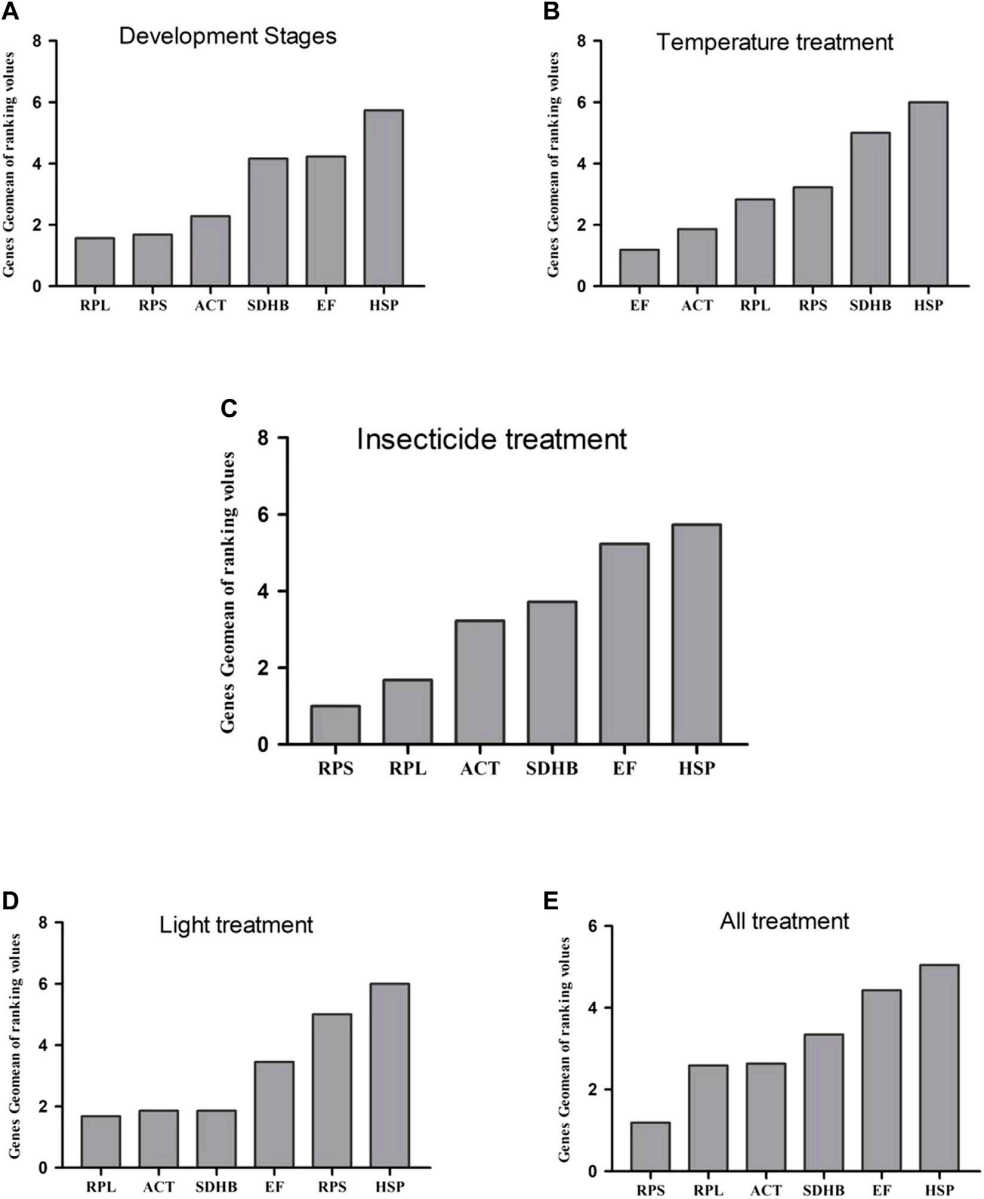
Expression stability of 6 reference genes in different samples. RefFinder geometric method was used to calculate the overall stability ranking of the reference genes. A lower Geomean of ranking value denotes more stable expression. **(A)** development stages; **(B)** insecticide; **(C)** temperature; **(D)** light; **(E)** The four treatments were analyzed simultaneously.

The total geomean values under different experimental conditions are shown in Figure 2. Expression in selected HKGs, except *HSP*, fluctuated slightly throughout the developmental stage (Figure 2A). Except for *HSP* and *SDHB,* expression in selected HKGs fluctuated slightly at different temperatures (Figure 2B). Under different insecticide treatments, *RPL* and *RPS* were the most stable, whereas *HSP* and *EF* were the least stable (Figure 2C). In addition to *HSP* and *RPS*, the expression of selected HKGs fluctuated slightly under light treatment (Figure 2D). All the results showed that *RPS, RPL,* and *ACT* had small changes, whereas *HSP* and *EF* had a large range of changes (Figure 2E).

### 3.3 Stability analysis of reference genes

**FIGURE 3:**
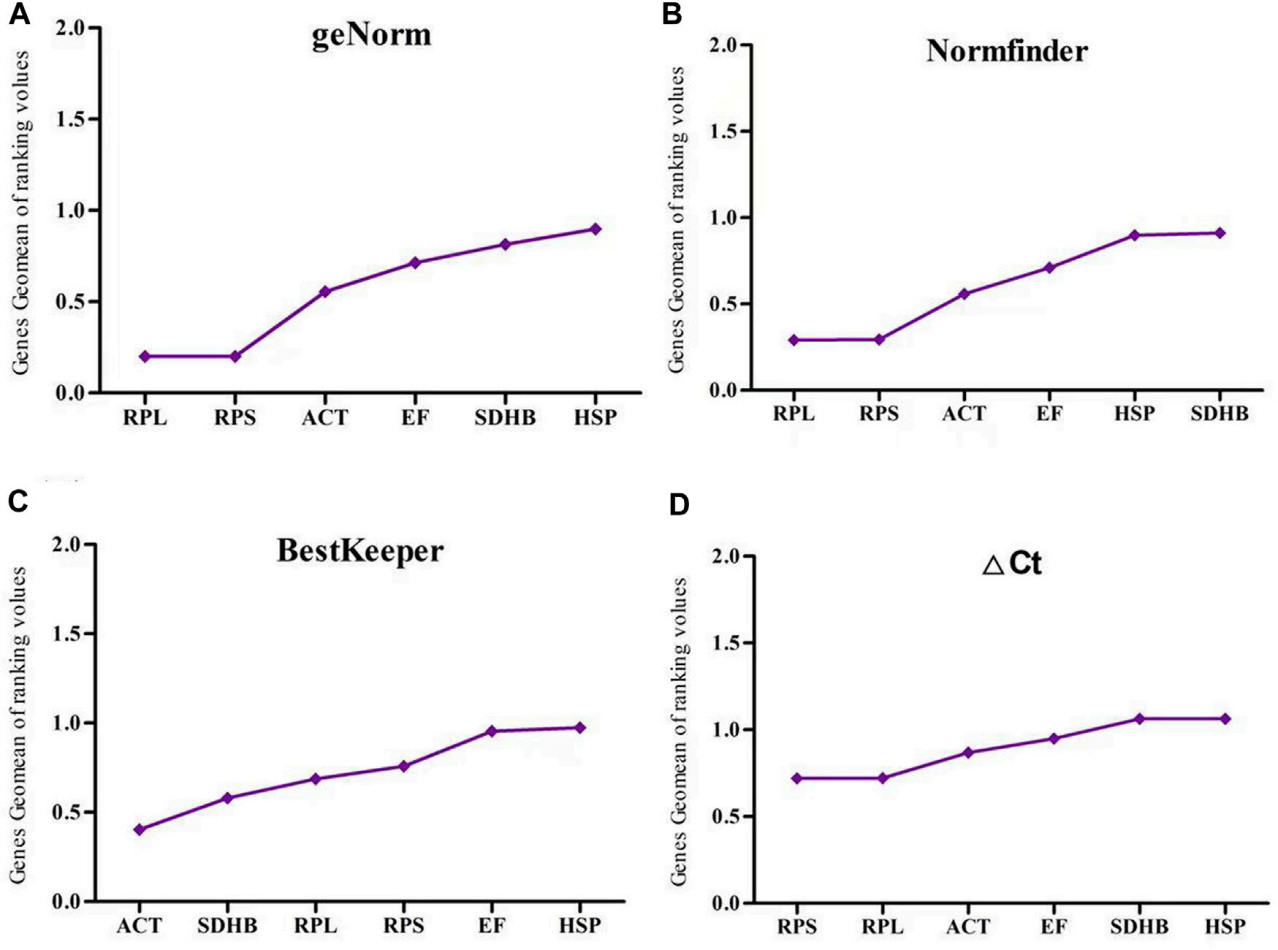
The stability of six reference gene different developmental stages samples of *Megalurothrips usitatus* genescalculated by the Geomean method of RefFinder. **(A)**, geNorm; **(B)**, NormFinder; **(C)**, BestKeeper; **(D)**, ΔCt.

RefFinder was used to analyze the expression stability of six reference genes under four experimental treatments. In the developmental stage treatment group, *ACT, RPS* and *RPL* were recommended as the most stable genes by geNorm, ΔCt and Normfinder, whereas *SDHB, ACT* and *RPL* were the most stable genes identified by BestKeeper. All four assays identified *HSP* as the most unstable gene (Figure 3).

**FIGURE 4:**
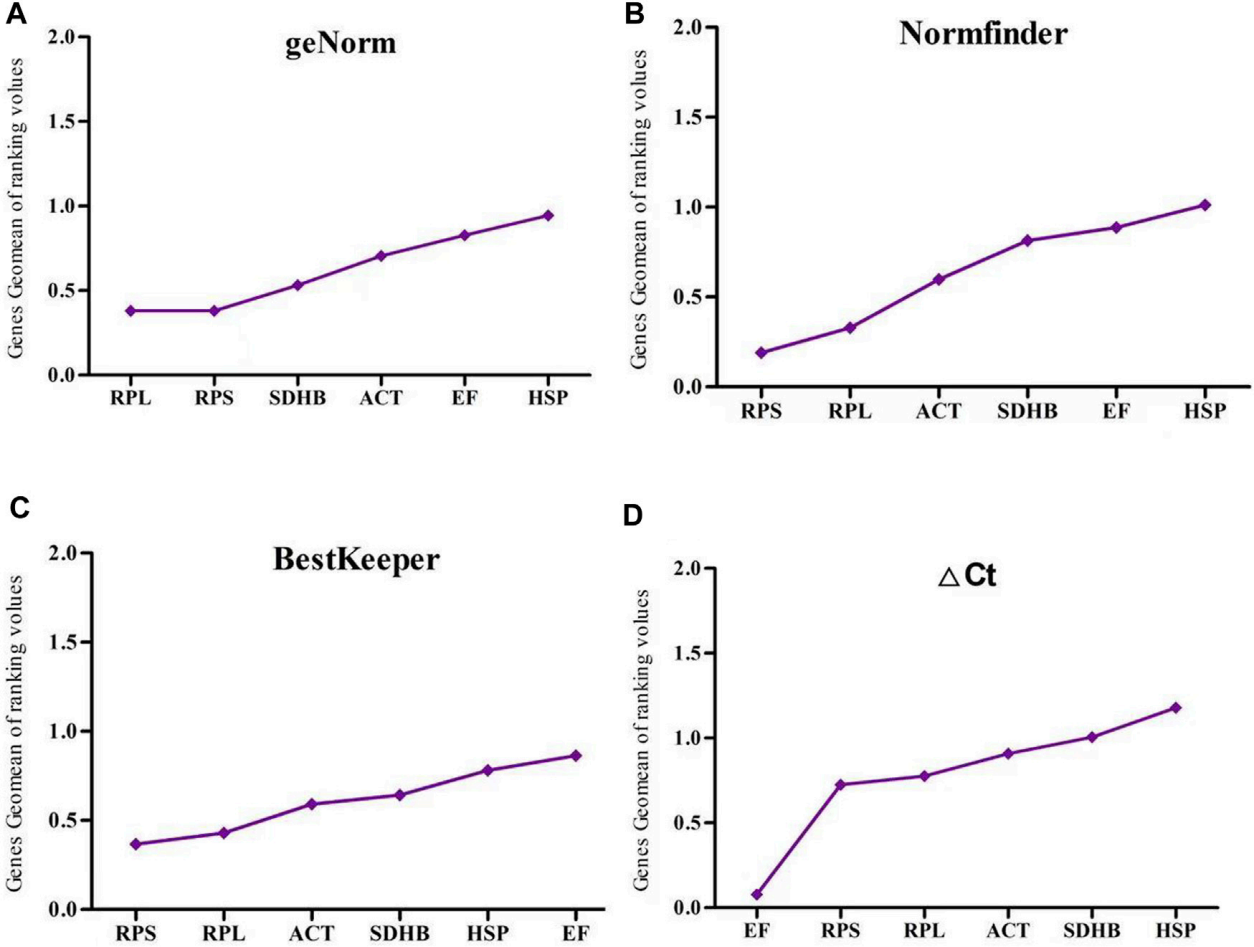
The stability of six reference gene different insecticide treatment samples of *Megalurothrips usitatus* genescalculated by the Geomean method of RefFinder. **(A)**, geNorm; **(B)**, NormFinder; **(C)**, BestKeeper; **(D)**, ΔCt.

In the insecticide treatment group, Normfinder and BestKeeper showed that *RPL*, *ACT*, and *RPS* were the most stable genes. ΔCT identified *RPS, RPL*, and *EF* as the most stable genes; geNorm identified *RPL, RPS*, and *SDHB* as the most stable genes; and all four analytical methods identified *HSP* as the most unstable gene (Figure 4).

**FIGURE 5:**
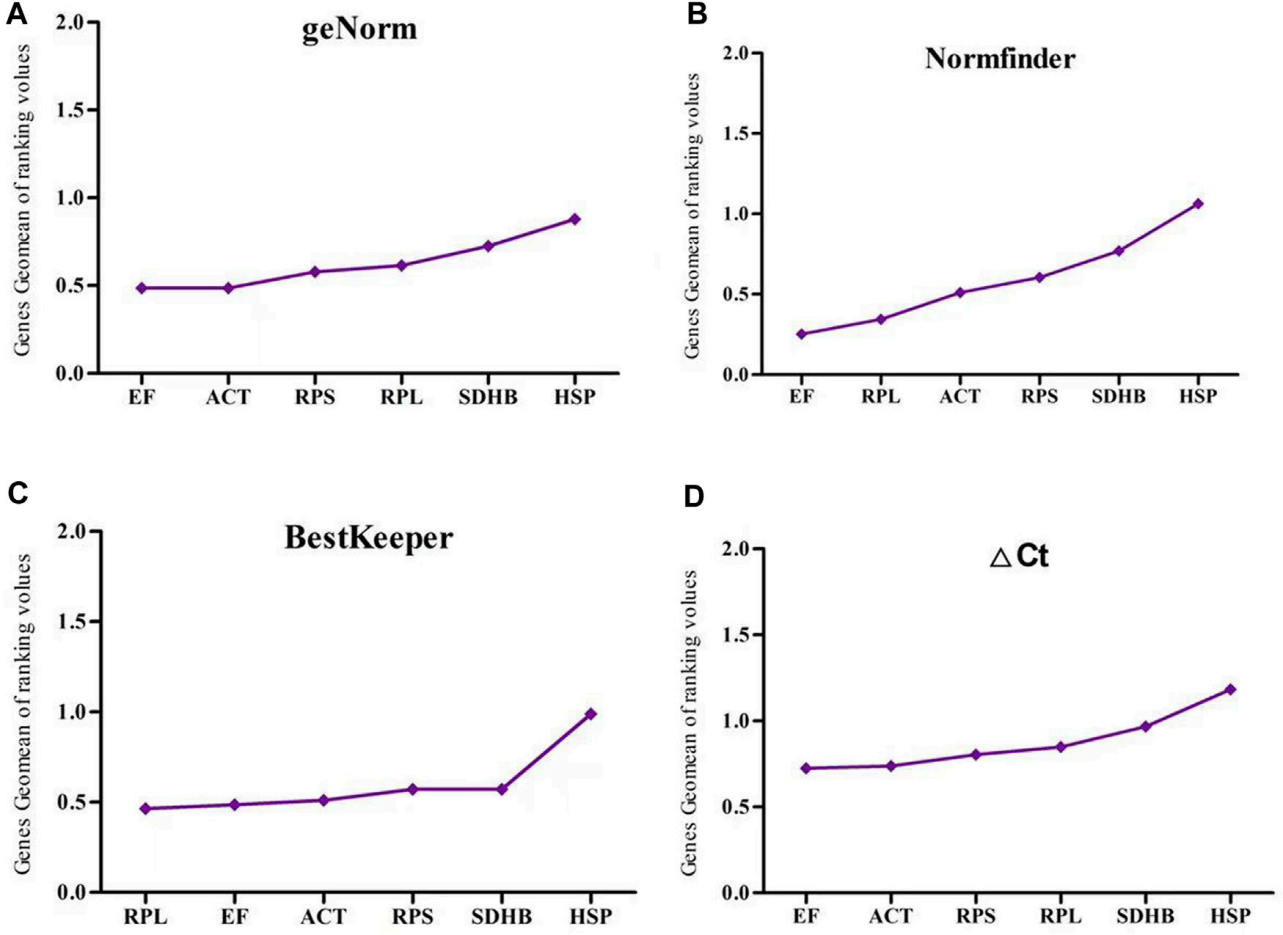
The stability of six reference gene different temperature treatment samples of *Megalurothrips usitatus* genescalculated by the Geomean method of RefFinder. **(A)**, geNorm; **(B)**, NormFinder; **(C)**, BestKeeper; **(D)**, ΔCt.

In the temperature treatment group, Normfinder and BestKeeper showed that *RPL, ACT*, and *EF* were the most stable genes. GeNorm and ΔCT identified *RPS, ACT*, and *EF* as the most stable genes. All four assays recommended *HSP* as the most unstable gene (Figure 5).

**FIGURE 6:**
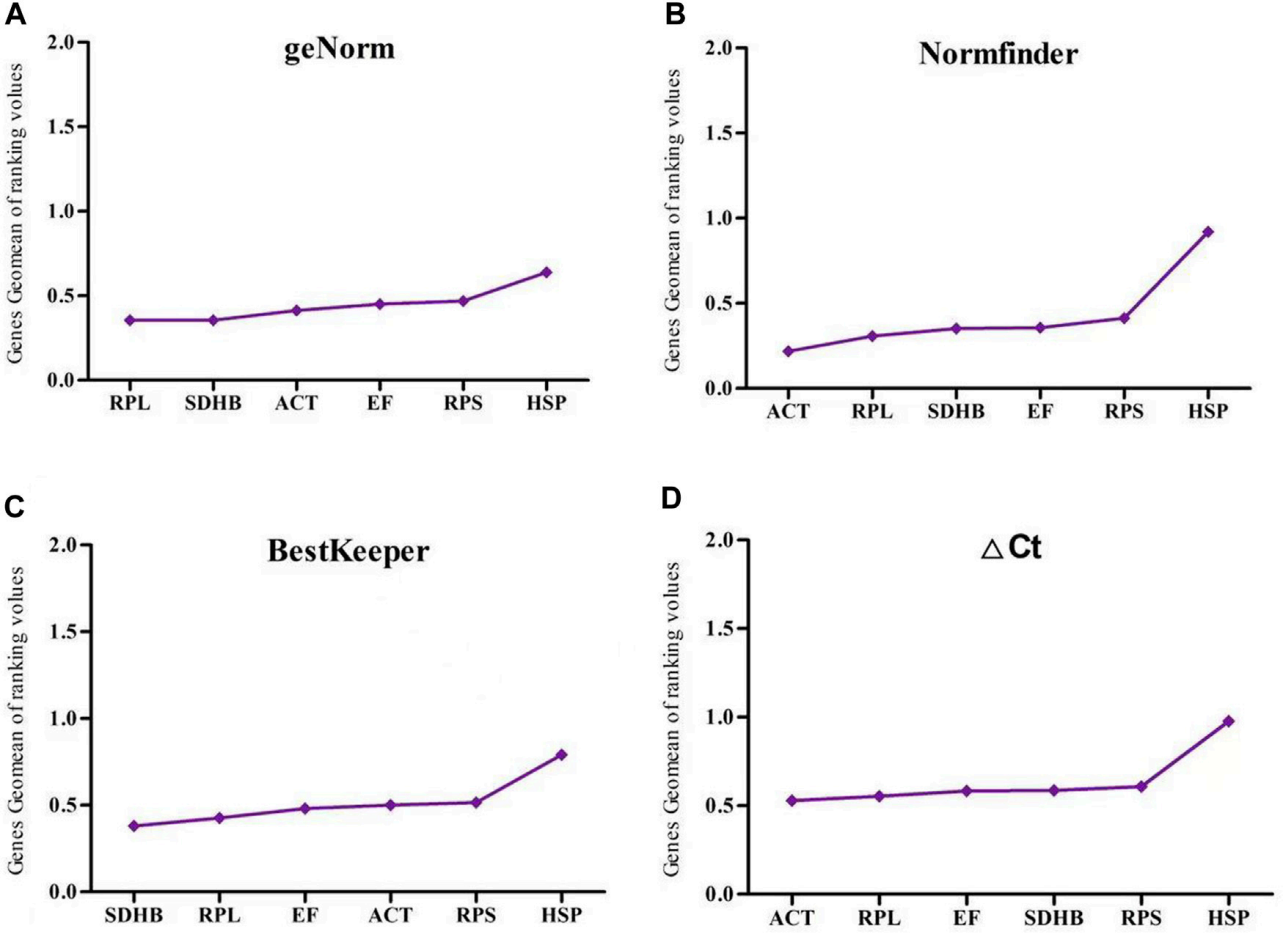
The stability of six reference gene different light treatment samples of *Megalurothrips usitatus* genescalculated by the Geomean method of RefFinder. **(A)**, geNorm; **(B)**, NormFinder; **(C)**, BestKeeper; **(D)**, ΔCt.

In the light treatment group, Normfinder and geNorm identified *RPL, ACT*, and *SDHB* as the most stable genes. BestKeeper identified *RPL, SDHB*, and *EF* as the most stable genes. ΔCt identified *RPL, ACT*, and *EF* as the most stable genes. Together, four analytical methods identified *RPS* and *HSP* as the most unstable genes (Figure 6).

In summary, according to RefFinder’s suggestion, the expression stability of reference genes in different developmental stages, insecticide, temperature, light and other treatments from the most stable to the most unstable is in the following order: *RPL > RPS > ACT > SDHB > EF > HSP; RPS > RPL > ACT > SDHB > EF > HSP; EF > ACT > RPL > RPS > SDHB > HSP; RPL > ACT > SDHB > EF > RPS > HSP* (Table 3).

**TABLE 3:** Expression stability of the reference genes under different experimental conditions.

Gene name	Stages of development		Temperature treatment		Insecticide treatment		Light treatment	
	Stability	Rank	Stability	Rank	Stability	Rank	Stability	Rank
*EF*	4.229	5	1.189	1	5.233	5	3.454	4
*RPL*	1.565	1	2.828	3	1.682	2	1.682	1
*ACT*	2.28	3	1.861	2	3.224	3	1.861	2
*RPS*	1.682	2	3.224	4	1	1	5	5
*SDHB*	4.162	4	5	5	3.722	4	1.861	3
*HSP*	5.733	6	6	6	5.733	6	6	6

The expression stability of candidate reference genes was analyzed in the aggregate data of various treatment experiments. According to RefFinder, the stability rankings for all samples (from most stable to most unstable) were as follows: *RPS > RPL > ACT > EF > SDHB > HSP.* Combining the results of the light and temperature treatments showed that *RPS* was unstable in light and temperature treatment; therefore, *RPL* and *ACT* were identified as the most stable reference genes of all treatments.

### 3.4 Validation of reference gene selection

**FIGURE 7:**
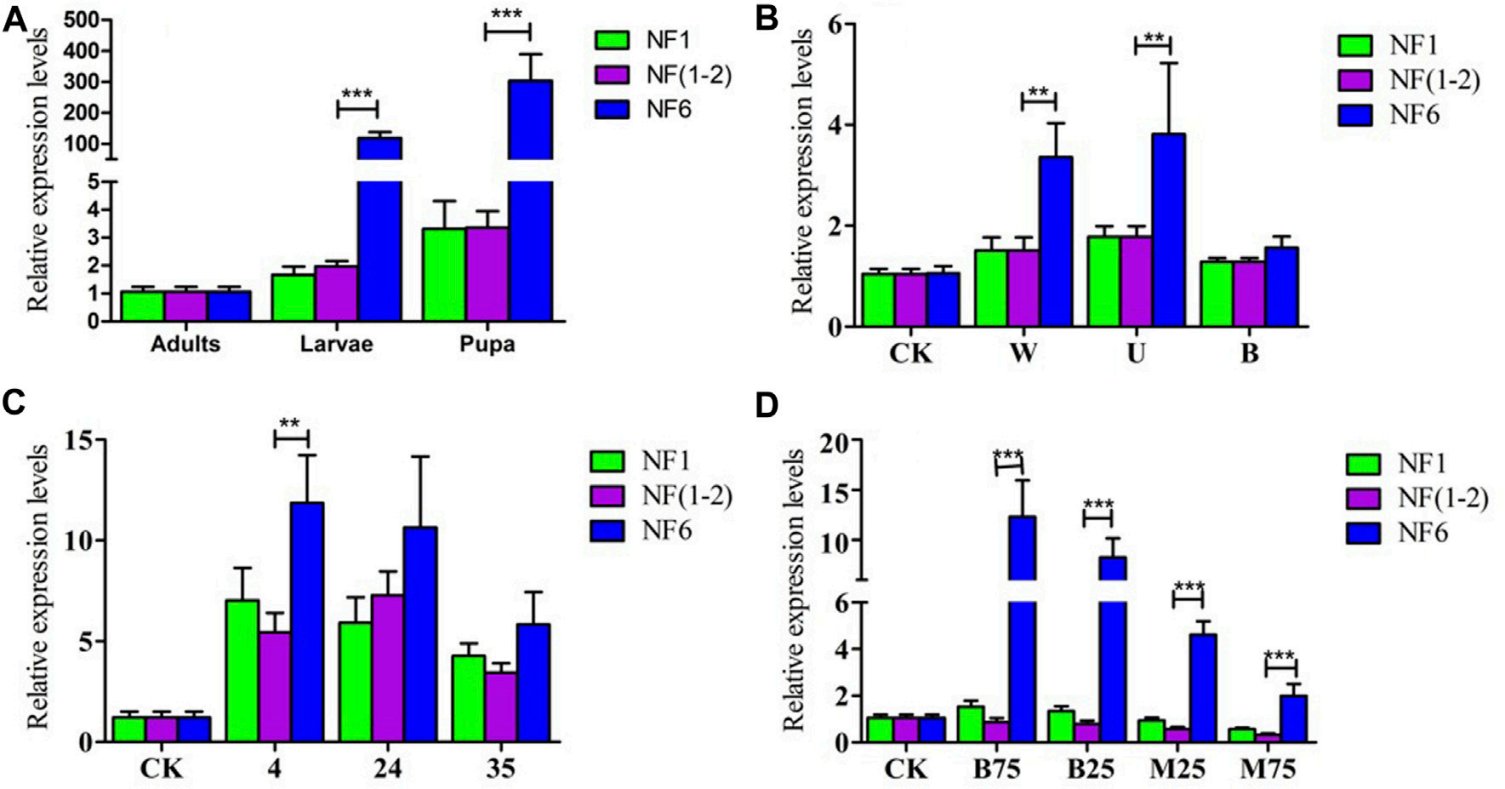
Relative expression levels of a target gene of interest (CYP450) were calculated using different sets of reference genes. **(A)** Different expression levels in three developmental stages, **(B)** different expression levels in three different light treatment, **(C)** different expression levels in varying temperature treatments, **(D)** different expression levels in varying insecticide treatments. The data represent the mean values ± SD. Bars represent the means and standard deviations of three biological replicates.

To assess the performance of selected reference genes, the expression level of *CYP450* was analyzed in the same experimental conditions used for the comparisons of the expression stability of the reference genes. The similar expression levels were obtained in the develop-mental stage experiments when normalized using the NF 1 (*EF*), the combination of the NF (1-2) (*EF* and *ACT*), However, when normalized with theNF6 (*HSP*), Significant differences were found in the expression level of *CYP450* in larvae and pupae (*p* < 0.001) (Figure 7A). Similar expression levels of *CYP450* were observed when normalized using the NF 1 (*RPL*), NF (1-2) (*RPL* and *ACT*) under different light treatment stages. However, when normalized with the NF6 (*HSP*), Significant differences were found in the expression level of *CYP450* in blue light and white light (*p* < 0.01) (Figure 7B). In different temperature treatment, the expression profiles of *CYP450* were significantly different at the different temperatures, no matter whether the NF1 (*EF*), the combination of the NF (1-2) (*EF* and *ACT*) or the NF6 (*HSP*) was used for the normalization. The *CYP450* expression levels were lower in the treatment groups by 35°C than by the 4°C–24°C (Figure 7C). In different insecticide treatment, When the NE6 (*HSP*) was normalized, its expression level was significantly higher than that of the NF1 (*RPS*), NF (1-2) (*RPS* and *RPL*) (*p* < 0.001). The *CYP450* expression levels were lower in the treatment groups by methylamectin benzoate than by the bifenthrin (Figure 7D).

## 4 Discussion

qRT-PCR technology provides a leap from qualitative to quantitative gene expression detection with the advantages of speed, efficiency, easy reproducibility, accurate quantification, high sensitivity, and high throughput (Su et al., 2013). It is a common technique in molecular biology research. In the analysis process, reference genes are often used to reduce or correct the errors in the quantitative process of target genes. Therefore, the selection of an appropriate reference gene is key to realizing the research on target gene expression under different experimental conditions or tissues. *M. usitatus* are an important pest of melon and vegetable crops; previous research has been limited to biological aspects, but it did not focus on their molecular function or gene expression. This work will benefit future studies on gene function researches of M. usitatus and other insects.

However, with the widespread use of qRT-PCR and gene chips, reports of reference genes are growing (Omondi et al., 2015). So far, reference genes for several insects have been selected and validated under different biological and abiotic conditions. These insects include *Apis mellifera* (Campbell EM, et al., 2016), *Bombyx mori* (Wang et al., 2008), *Schistocerca gregaria* (Van Hiel et al., 2009), *Tribolium castaneum* (Lord et al., 2010), *Drosophila melanogaster* (Lordet al., 2011), *Agrilus planipennis* (Priya et al., 2012), *Plutella xylostella* (Fu et al., 2013), *Bemisia tabaci* (Yang et al., 2021), *Solenopsis invicta* (Cheng et al., 2013), *Helicoverpa armigera* (Yang et al., 2014), and *Spodoptera exigua* (Zhu et al., 2014). The results show that no gene can be expressed constantly under complex experimental conditions. Previous studies have shown that two or three reference genes should be used to ensure more accurate results, and different combinations of reference genes should be used under different experimental conditions (Zhang et al., 2015). Therefore, before qRT-PCR experiments, reference genes that are stably expressed in specific experimental materials conditions must be screened out.

In this study, insects were selected to select the commonly used endogenous reference gene in *M. usitatus.* It was evaluated for stability at different developmental stages, light, temperature, and insecticide treatments at different time. This study is the first to evaluate and verify candidate reference genes of *M. usitatus. EF, ACT, HSP, SDHB, RPS,* and *RPL* were found to have high consistency with the corresponding gene sequences of other insects, and the matching difference was small, *RPL* and *ACT* were the best candidate for the reference gene. This result further proved that these reference genes were highly conserved in insect evolution.

The screening of reference genes usually uses professional software to analyze the stability of reference genes, and used to analyze the stability of the tested reference genes by calculating their geometric mean for the overall ultimate ranking. Numerous studies have shown that the combination of multiple reference genes is more advantageous than a single gene, but too many reference genes cause unnecessary experimental data and make subsequent gene expression calculations more cumbersome; thus, the optimal number of reference genes is usually two (Majerowicz et al., 2011). To avoid the limitations of using only a single software analysis, four analytical methods were used in this study. The rankings from different programs showed some substantial discrepancies. Therefore, to provide a comprehensive evaluation of candidate reference genes, geometric mean was used to generate a comprehensive stability ranking, and the top two stable values of stability were given in the analysis of different conditions. Thus, two reference genes are the ideal number in this study, which is consistent with the suggestions of many scholars. The results show that the expression stability of the six most used reference genes (*EF*, *ACT*, *RPL*, *RPS*, *SDHB*, *HSP*) in *M. usitatus* varies, and the stability ranking of reference genes also differs under different experimental conditions.

In this study, *RPL* and *RPS* were the most stable reference genes throughout the developmental stage and in insecticide experiments. Similar results were obtained in *Bactrocera minax* (Chen et al., 2014) and *Cimex lectularius* (Zhang et al., 2018). Stable expression was also achieved in *H. armigera* treatments, whereas RPS was identified as the most unstable gene in light treatment experiments. In addition, *ACT* and EF were the most stable genes expressed in temperature treatment in this study; *ACT* also showed stable expression in the developmental stage of *Eri-silkworm*, *D. melanogaster* temperature treatment, and *EF* was identified as the most unstable gene in pharmacy treatment and developmental experiments. In other species, such as *Bombus lucorum*, *Chortoicetes terminifera* and *B. tabaci*, *EF* was identified as a reference gene for stable expression. *RPL* and *SDHB* were the most stable genes in light treatment experiments, whereas *SDHB* is the most unstable gene in temperature treatment. Therefore, the selection of reference genes varies in accordance with differences in species or experimental conditions, and the most suitable reference genes are often different between different species.

To the best of our knowledge, this study is the first systematic report on the selection and verification of reliable stable reference genes for different tissues in *M. usitatus*. Results show that among these six reference genes, the expression levels of *RPL* and *ACT* vary minimally under various experimental conditions. This study provides a basis for gene expression in *M. usitatus*.

Permission must be obtained for use of copyrighted material from other sources (including the web). Please note that it is compulsory to follow figure instructions.

## Data Availability

The original contributions presented in the study are included in the article/supplementary material, further inquiries can be directed to the corresponding authors.
