# Corrigendum: Identification and validation of reference genes for normalization of gene expression analysis using qRT-PCR in *Megalurothrips usitatus* (thysanoptera: thripidae)

**DOI:** 10.3389/fphys.2025.1612671

**Published:** 2025-04-25

**Authors:** Qingfang Hou, Linlin Yuan, Haifeng Jin, Han Yan, Fen Li, Shaoying Wu

**Affiliations:** ^1^ Sanya Nanfan Research Institute, Hainan University, Sanya, China; ^2^ School of Plant Protection, Hainan University, Haikou, China

**Keywords:** *Megalurothrips usitatus*, light, qRT-PCR, reference genes, stability

In the published article, Xie, F., Xiao, P., Chen, D., Xu, L., Zhang, B. (2012). miRDeepFinder: a miRNA analysis tool for deep sequencing of plant small RNAs. Plant Mol. Biol. 80, 75–84. doi: 10.1007/s11103-012-9885-2 was not cited. The citation has now been inserted in **2 Materials and methods**, *2.6 Stability analysis of reference gene expression*, Paragraph 1 and should read:

“A web-based analysis tool, namely, RefFinder (https://www.heartcure.com.au/reffinder), was used to conduct a comprehensive assessment on the stability of the candidate. RefFinder integrated all the four software applications, including geNorm, NormFinder, BestKeeper, and compared ΔCt method. On the basis of the rankings from each program, RefFinder assigned an appropriate weight to an individual gene and calculated the geometric mean of their weights for the overall final ranking **(Xie et al., 2012)**.”

The authors apologize for this error and state that this does not change the scientific conclusions of the article in any way. The original article has been updated.

