# Supplementary figures and images for: Identification and validation of reference genes for normalization of gene expression analysis using qRT-PCR in Megalurothrips usitatus (thysanoptera: thripidae)
Source: Front Physiol. 2023 Apr 18;14:1161680. doi: 10.3389/fphys.2023.1161680 (PMC10151585; doi:10.3389/fphys.2023.1161680)

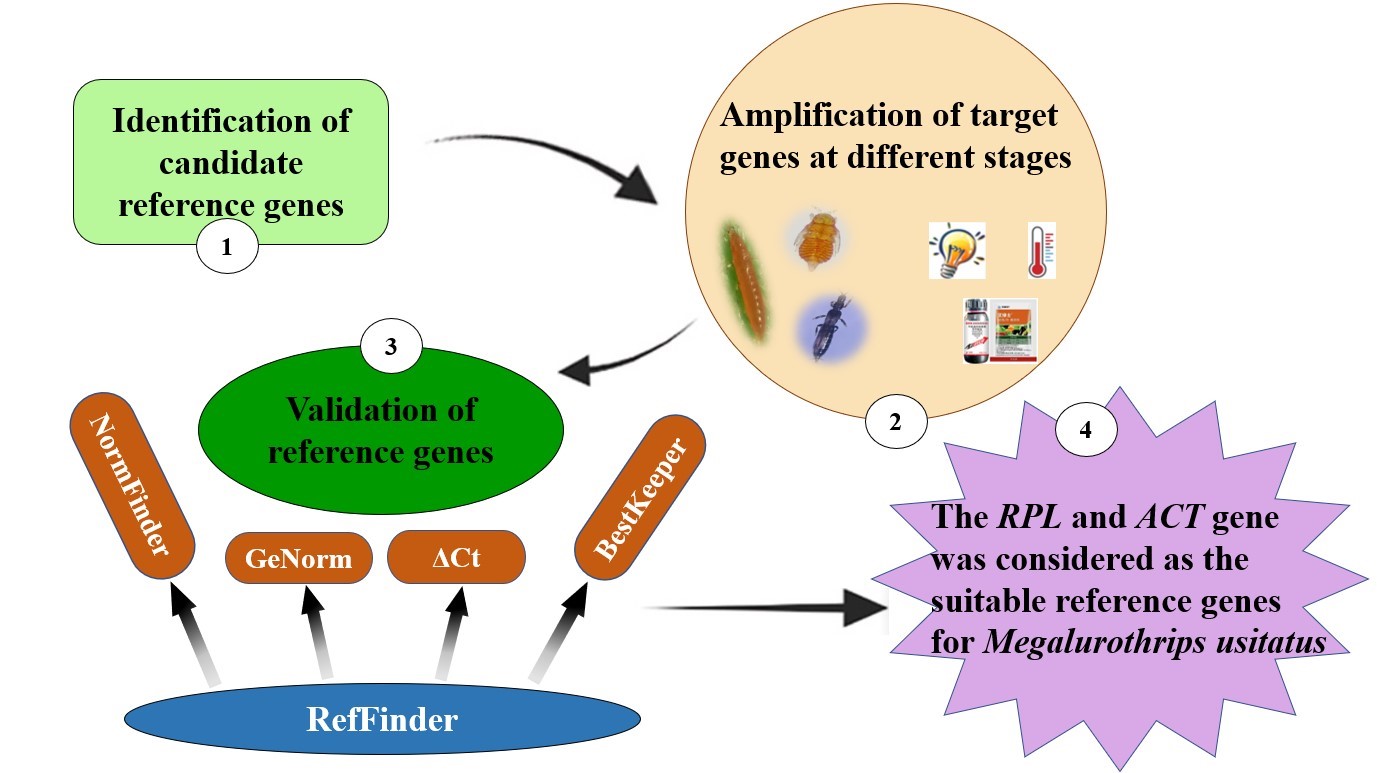

Supplement: Supplementary file 1 [file Image1.JPEG]
